# Size-independent symmetric division in extraordinarily long cells

**DOI:** 10.1038/ncomms5803

**Published:** 2014-09-15

**Authors:** Nika Pende, Nikolaus Leisch, Harald R. Gruber-Vodicka, Niels R. Heindl, Jörg Ott, Tanneke den Blaauwen, Silvia Bulgheresi

**Affiliations:** 1 Department of Ecogenomics and Systems Biology, University of Vienna, Althanstrasse 14, 1090 Vienna, Austria; 2 Department of Symbiosis, Max Planck Institute for Marine Microbiology, Celsiusstrasse 1, D-28359 Bremen, Germany; 3 Department of Limnology and Biooceanography, University of Vienna, Althanstrasse 14, 1090 Vienna, Austria; 4 Department of Bacterial Cell Biology, Swammerdam Institute of Life Sciences, Faculty of Science, University of Amsterdam, Science Park 904, 1098 XH Amsterdam, the Netherlands; 5 Center for Anatomy and Cell Biology, Medical University of Vienna, Währingerstrasse 10, 1090 Vienna, Austria; 6 These authors contributed equally to this work

## Abstract

Two long-standing paradigms in biology are that cells belonging to the same population exhibit little deviation from their average size and that symmetric cell division is size limited. Here, ultrastructural, morphometric and immunocytochemical analyses reveal that two *Gammaproteobacteria* attached to the cuticle of the marine nematodes *Eubostrichus fertilis* and *E. dianeae* reproduce by constricting a single FtsZ ring at midcell despite being 45 μm and 120 μm long, respectively. In the crescent-shaped bacteria coating *E. fertilis*, symmetric FtsZ-based fission occurs in cells with lengths spanning one order of magnitude. In the *E. dianeae* symbiont, formation of a single functional FtsZ ring makes this the longest unicellular organism in which symmetric division has ever been observed. In conclusion, the reproduction modes of two extraordinarily long bacterial cells indicate that size is not the primary trigger of division and that yet unknown mechanisms time the localization of both DNA and the septum.

## Abstract (web summary)

Known mechanisms that determine symmetric division-plane positioning during cell division are unlikely to operate effectively over very long distances. Pende *et al*. show that extraordinarily long Gammaproteobacteria divide symmetrically despite reaching 120 microns in length

## Graphical abstract

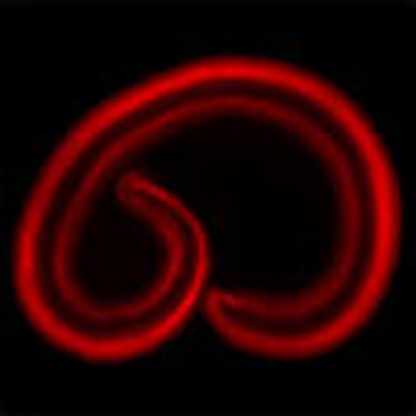

The spatial control of bacterial growth, division and morphology—as well as the mechanisms of DNA organization and segregation—has been investigated only in a handful of cultivable organisms so far^1^. Given the overwhelming phylogenetic and morphological diversity of microbes, our current knowledge on their cell biology likely represents only a tiny piece of the overall picture. This is regrettable given that the void in antibiotics discovery^2^, coupled with an increasing awareness about the role of uncultivable, non-pathogenic microbes in animal health, implies major applied benefits from studying how environmental bacteria reproduce.

Most known bacteria are between 0.4 and 2 μm in diameter, and 0.5 and 5 μm in length. Model rod-shaped bacteria control their overall size by varying their cell length, which, in turn, is under control of the division apparatus^3^,^4^. *Escherichia coli*, for example, can grow into long, aseptate filaments with regularly spaced nucleoids following mutations in filamentous temperature sensitive (*fts*) genes, which direct the assembly of the division apparatus^5^. Although these *E. coli* mutants cannot build a functional constricting ring and finally lyse, DNA replication and chromosome segregation continue unaffected for several generations.

The metabolic status of the cell may also affect bacterial length: *Bacillus subtilis* is longer when incubated in a nutrient-rich medium and shorter when nutrients are limited^6^,^7^. In the former case, *B. subtilis* accumulates a metabolite that induces a glucosyltransferase. This, in turn, inhibits FtsZ polymerization so that cell division is delayed and the cells grow longer^8^. In a striking example of convergent evolution, the size of *E. coli* is similarly linked to nutrient availability by a different but functionally analogous glucosyltransferase^9^,^10^,^11^,^12^.

With the sole exception of the giant surgeonfish gut symbiont^13^, the molecular basis of the reproduction of naturally occurring long to giant bacteria (longest length between 10 and 750 μm) is unexplored. Many of them are marine sulphur-oxidizing bacteria (SOBs) and contain large nitrate and sulphur inclusions that reduce the volume of their active cytoplasm^14^. Here we investigated the molecular mechanisms underlying the reproduction mode of two extraordinarily long bacteria coating the newly described nematode species *Eubostrichus fertilis* (Fig. 1a–d and Supplementary Fig. 1a)^15^ and *E. dianeae*^16^,^17^ (Supplementary Fig. 1b; hereafter we refer to these filamentous bacteria as Efs and Eds, respectively). Eds contains strongly refracting spherical cytoplasmic inclusions^17^ and Raman microspectrometry proved that at least some of these contain elemental sulphur^18^,^19^. Although these data suggest that Eds may store and oxidize sulphur, previous 16S ribosomal RNA (rRNA) gene-based analyses of the *E. dianeae*-associated bacterial community did not yield any SOB 16S rRNA gene sequence^20^. As for *E. fertilis*, crescent-shaped bacteria are attached with both poles to their nematode host so that their long axis is always parallel to the host’s anterior–posterior axis. This, combined with their shifted alignment to one another around the worm circumference, confers a rope-like appearance (Fig. 1a), similar to that observed for *E. cf. parasitiferus*^16^,^17^, *E. topiarius*^21^ and *Adelphus rolandi*^22^. Although DNA staining of *E. cf. parasitiferus*-associated bacteria revealed several nucleoids in each cell^17^, binary fission was detected neither in these, nor in Eds cells. It was therefore hypothesized that both ectosymbionts elongate without dividing, possibly due to nematode-secreted inhibitors^17^.

This study was designed to determine how supersized bacteria associated with two *Eubostrichus* nematodes reproduce on their respective hosts.

## Results

### One bacterial phylotype coats each nematode species

To molecularly identify the filamentous bacteria coating *E. fertilis* and *E. dianeae*, we extracted genomic DNA (gDNA) from single symbiotic worms and constructed bacterial 16S rRNA gene libraries. Based on BLASTN search, we identified three and seven SOB 16S rRNA sequences in the *E. fertilis* and *E. dianeae* libraries, respectively. We compared these SOB sequences to those of other stilbonematid and oligochaete symbionts, as well as to those of bacteria belonging to the *Chromatiaceae* and other uncultured *Gammaproteobacteria* that are >95% similar to the symbionts. The resulting 16S rRNA gene-based phylogenetic tree (Fig. 2) shows that the representative SOB sequences, each obtained either from the *E. fertilis* or the *E. dianeae* 16S rRNA gene library (GenBank accession numbers KF278590 and KF278587, respectively) belong to the Marine Oligochaete and Nematode Thiotrophic Symbionts (MONTS) cluster of *Gammaproteobacteria*^23^ and are most closely related to *E. topiarius*-associated bacteria. To confirm that the MONTS 16S rRNA sequences obtained in our libraries originated from Efs and Eds, we applied fluorescence *in situ* hybridization (FISH) probes specifically targeting those sequences (probe Efs1057 and Eds214, respectively) to whole mount *Eubostrichus* nematodes. All bacteria attached to the host surface were triple stained by the bacterial probe EUB338, by the *Gammaproteobacteria*-specific probe GAM42a and by the respective MONTS-specific probes (Fig. 3 and Supplementary Fig. 2). This confirms that the MONTS 16S rRNA gene sequences obtained in our libraries originated from the filamentous bacteria coating *E. fertilis* and *E. dianeae*. Moreover, given that all bacteria detected by the *Eubacteria*-specific probe were also detected by the *Gammaproteobacteria*- and MONTS-specific probe, the non-MONTS 16S rRNA gene sequences identified in our libraries, including those attributable to *Deltaproteobacteria* (Supplementary Fig. 2) did not originate from cuticle-associated bacteria. In conclusion, Efs and Eds are two novel distinct MONTS phylotypes, each one associated with the respective host species. The phylogenetic placement of Eds and Efs is consistent with that observed for all marine nematode ectosymbionts characterized by full 16S rRNA-gene cycle so far^24^,^25^,^26^. Moreover, given that Efs and Eds were the only bacteria detected on the surface of their respective hosts, these newly characterized stilbonematid ectosymbioses also appear to be monospecific.

### The nematode symbionts express the tubulin homologue FtsZ

In the model gammaproteobacterium *E. coli*, cell division is initiated by polymerization of the tubulin homologue FtsZ into a ring (the Z-ring) and its subsequent constriction^27^,^28^,^29^. In *E. coli* cells, the ring is usually positioned at midcell, perpendicular to the longitudinal axis^30^. After the self-assembly the Z-ring starts to constrict, thereby directing the division of the cell, which results in two equal daughter cells. Given that FtsZ mediates binary fission in all known *Gammaproteobacteria*, including the longitudinally dividing MONTS associated with *Laxus oneistus*^31^, and it is part of the genetic repertoire of three additional MONTS (genome drafts available upon request at http://rast.nmpdr.org/rast.cgi), we hypothesized that Efs and Eds also express the *fts*Z gene. By using degenerate PCR primers on gDNA extracted from single symbiotic nematodes, we homology cloned Efs and Eds *ftsZ*-gene fragments. The predicted Efs FtsZ protein fragment (322 amino acids) had 83% sequence identity with that of the *L. oneistus ecto*symbiont, and 67% with that of *E. coli* K12, whereas the corresponding sequence identities for the predicted Eds FtsZ protein fragment (336 amino acids) were 84% and 66%, respectively. The *Eubostrichus* symbiont FtsZ proteins cluster with other MONTS FtsZ proteins in our phylogenetic reconstruction (Supplementary Fig. 3). To assess if the *Eubostrichus* ectosymbionts express FtsZ, a commercially available anti-*E. coli* FtsZ antibody was tested on Western blots of protein extracted from host-dissociated ectosymbionts. This resulted in the specific detection of protein bands of ~40 kDa, the predicted molecular weight of *E. coli* FtsZ (Supplementary Fig. 4). In conclusion, both Efs and Eds express the cell division protein FtsZ and their genes are phylogenetically related to the *L. oneistus* ectosymbiont FtsZ and form a 16S rRNA gene-concordant cluster.

### Symmetric FtsZ-based fission in 4 to 45 μm-long Efs cells

**Figure 1:**
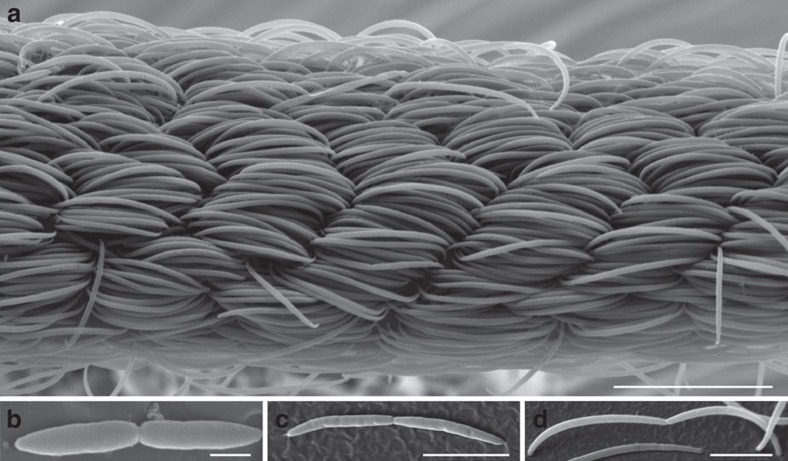
Scanning electron microscope (SEM) micrographs of the *E. fertilis* ectosymbiont. (**a**) Central portion of the bacterial coat and (**b**–**d**) single constricted Efs cells of different lengths. Scale bar, 20 μm in **a**, 1 μm in **b** and 5 μm in **c**,**d**.

**Figure 2:**
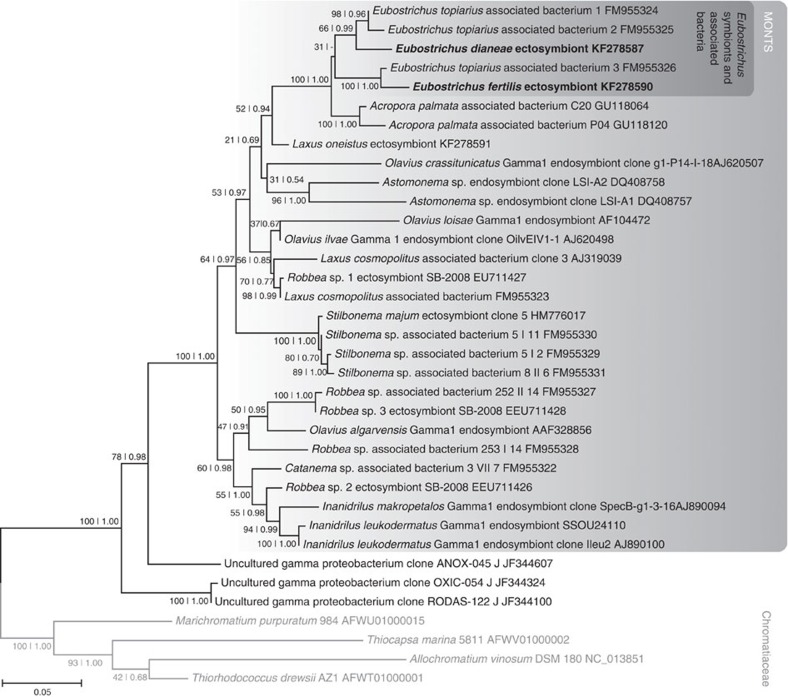
Gammaproteobacterial 16S rRNA gene tree based on the most likely RAxML tree (GTR+I+G model of substitution). Rapid bootstrapping (RAxML) and posterior probability (MrBayes) node support is given for all nodes. The minus symbol (−) instead of a posterior probability in the *Eubostrichus*-associated bacteria cluster indicates that this node was not resolved in the MrBayes consensus. The *Chromatiaceae* outgroup is indicated with grey font colour. Scale bar represents the mean number of nucleotide substitutions per site. GenBank accession numbers are indicated after the names of the sequences.

**Figure 3:**
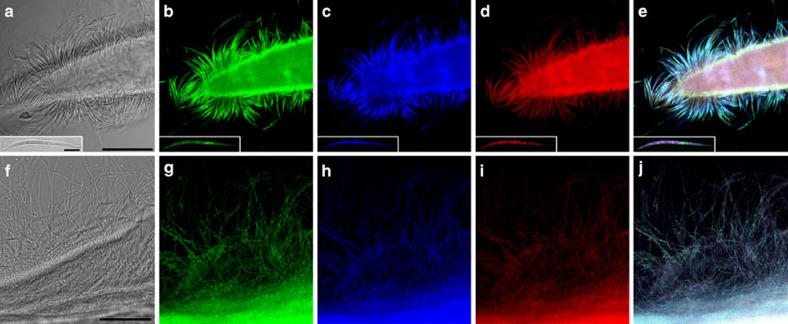
FISH/laser-scanning confocal microscopy (LSCM) of ectosymbionts attached to the worm surface. Images of the Efs coat are shown in **a**–**e** and images of the Eds coat in **f**–**j**. **a** and **f** are the corresponding bright field images of **b**–**e** and **g**–**j**, respectively. Each single symbiont is triple stained with specific probes targeting *Eubacteria* (**b** and **g**), *Gammaproteobacteria* (**c** and **h**), and the symbiont (Efs1027 and Eds214, respectively; **d** and **i**). (**e**) and (**j**) are overlay pictures of (**b**–**d**) and (**g**–**i**), respectively. A single Efs cell is shown in the insets in (**a**–**e**). Scale bar, 25 μm for all images and 5 μm for the insert.

**Figure 4:**
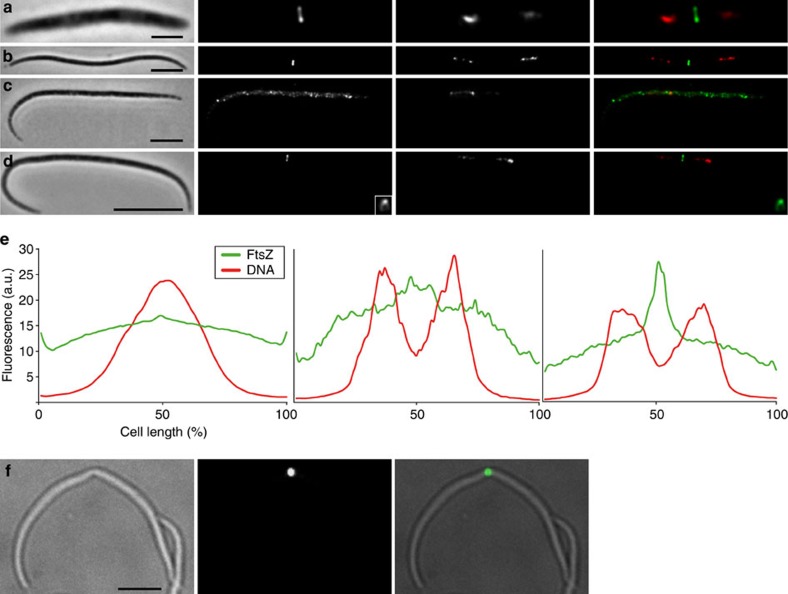
FtsZ and DNA localization pattern in Efs cells. (**a**–**d**) From left to right, phase contrast, FtsZ fluorescence, DNA fluorescence and FtsZ (green)/DNA (red) overlay images of immunostained Efs cells of different lengths (**a**,**b** and **d** have FtsZ rings and segregated DNA, **c** has neither mid-cell FtsZ accumulation nor segregated DNA). Insets in **d** show an LSCM image of an FtsZ ring from a different cell. **e** shows average fluorescence profiles of 549 cells displaying neither FtsZ mid-cell accumulation nor DNA segregation (left), 36 cells with both non-ring FtsZ accumulation at mid-cell (middle) and segregated DNA, and 77 cells with FtsZ rings and segregated DNA (right). FtsZ fluorescence (green) and DNA fluorescence (red) expressed in arbitrary units (a.u.) are plotted along the cell length expressed in percentage. LSCM images of an immunostained Efs constricted cell are shown in **f**, from left to right bright field, FtsZ fluorescence and overlay. Scale bars. 2 μm in **a**, 5 μm in **b**,**c** and **f** and 10 μm in **d**.

Morphometric analysis of 2,731 Efs cells showed a length range of 3.54–44.55 μm and a width range of 0.38–1.21 μm (Table 1; Supplementary Fig. 5) and scanning electron microscopy (SEM) analysis revealed constricted cells of lengths ranging from 6.1 to 23.2 μm (Fig. 1b–d). Consistently, Efs cell length distribution indicated that most length values are similarly represented in the population (Supplementary Fig. 5). To test the hypothesis that Efs cells can divide at any length, we immunostained them with an anti-FtsZ antibody and analyzed its localization pattern. FtsZ rings appeared in 77/662 cells (11.6%) between 3.5 and 45 μm long (Fig. 4a,b,d,f and Supplementary Fig. 6). Unsegregated DNA localized at midcell in cells with homogeneous FtsZ staining (Fig. 4e left plot and representative image in Fig. 4c), whereas DNA was already segregated in cells with mid-cell (non-ring) FtsZ accumulation (Fig. 4e, middle plot). In cells showing a Z-ring (e.g. insert Fig 4d insert), we observed symmetric DNA segregation both in terms of localization and of fluorescence signal intensity (Fig. 4e, rightmost plot). Of note, in these cells, the DNA signal was often highly fragmented as if originating from several nucleoids (see for example Fig. 4b or Supplementary Fig. 6b,n). Mid-cell membrane constriction in correspondence with mid-cell FtsZ signal indicates that FtsZ polymerized into a functional constricting ring (Fig. 4f). FtsZ fluorescence profiles of 662 cells aligned from the shortest to longest (left to right in Supplementary Fig. 7a–d) did neither indicate a continuous FtsZ fluorescence accumulation at midcell, nor a continuous DNA partitioning pattern as Efs lengthens. However, most of the cells displaying mid-cell FtsZ signal (62.3%; Supplementary Table 1) were 15–30-μm long. We conclude that Efs cells divide by FtsZ-based fission and may symmetrically segregate the DNA at virtually every length leading to a 10-fold variation in cell length.

### Symmetric FtsZ-based fission in up to 120-μm-long Eds cells

**Figure 5:**
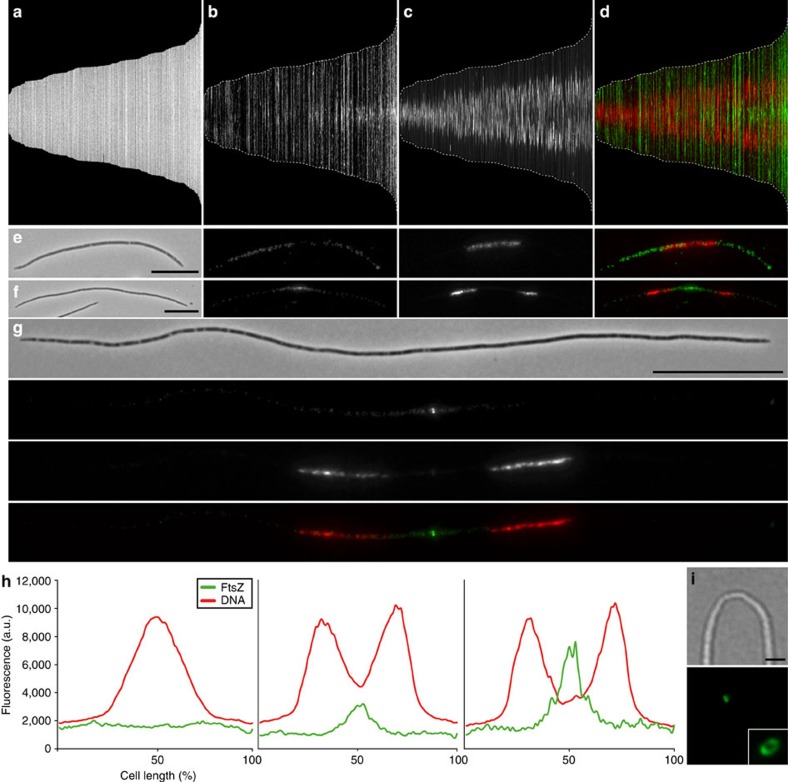
FtsZ and DNA localization pattern in Eds cells. Cell length, FtsZ fluorescence, DNA fluorescence and overlay of FtsZ (green) and DNA (red) fluorescence of 562 Eds cells are represented in **a**–**d**, respectively. Cells are sorted by increasing cell length from left to right that is, the leftmost bar represents the shortest and the rightmost bar the longest cell. Cell length ranges from 16.46 to 82.54 μm (mean 43.16±12.30). Dotted white line in **b**–**d** indicates the cell outline. (**e**–**g**) Phase contrast, FtsZ fluorescence, DNA fluorescence and FtsZ (green)/DNA (red) overlay images of immunostained Eds cells of different lengths displayed from left to right in **e** and **f** and top to bottom in **g**. **h** shows average fluorescence profiles of 301 cells with homogeneous FtsZ staining and non-segregated DNA (left), 189 cells with FtsZ accumulation at mid-cell and segregated DNA (middle), and 54 cells with FtsZ rings and segregated DNA (right). FtsZ fluorescence (green) and DNA fluorescence (red) expressed in arbitrary units (a.u.) are plotted along the cell length in percentage. LSCM images of a constricted immunostained Eds cell is shown in **i**, bright field and corresponding FtsZ fluorescence below. Inset shows an LSCM image of a FtsZ ring from a different cell. Scale bars, 10 μm in **e** and **f**, 20 μm in **g** and 2 μm in **i**.

Each Eds filament is attached to the nematode host by one of its poles (Supplementary Fig. 1b,d). Morphometric analysis of 2,743 Eds cells showed a length range 16.5–120.2 μm and a width range of 0.4–1.4 μm (Table 1), albeit most cells are ~50-μm long (Supplementary Fig. 5). To assess if Eds divides via FtsZ-based fission, we immunostained it with an anti-FtsZ antibody and analyzed its localization pattern. In the shortest cells, FtsZ was homogenously distributed throughout the cell, whereas unsegregated DNA localized at midcell (Fig. 5a–d, the shortest cells correspond to the bars occupying—approximately—the leftmost third of the profile; representative single cell in Fig. 5e and leftmost plot in h). In the medium length cells, FtsZ fluorescence accumulated in a central portion of the cell between the segregated DNA (central third of each profile in Fig. 5a–d, representative image in f and central plot in h). In the longest cells, FtsZ appeared as a sharp, mid-cell band and DNA was further segregated into the two prospective daughter cells (rightmost third of each profile in Fig. 5a–d, representative image in g and rightmost plot in h). Notably, and as observed for Efs, only a small fraction of the Eds cells displayed FtsZ rings (87/772, 11.27%). However, all of them were longer than 30 μm, indicating that the propensity of Eds to divide increases as it lengthens (Supplementary Table 1). Plotting the DNA fluorescence emitted by 301 cells with non-segregated DNA (representative image in Supplementary Fig. 8b) and by 237 cells with segregated DNA (representative image in Supplementary Fig. 8c) against the cell length confirmed symmetric partitioning of the DNA into the two prospective daughter cells (Supplementary Fig. 8a). As observed in Efs cells displaying FtsZ rings, the DNA signal was often highly fragmented as if originating from several nucleoids (Supplementary Fig. 8c). Further, confocal microscopy revealed that mid-cell FtsZ polymerizes into a circular ring in Eds and that this is functional as it can drive cell membrane constriction (Fig. 5i). We conclude that the gammaproteobacterium Eds, despite its extraordinary length, divides transversally and symmetrically by means of a single, constricting FtsZ ring placed at midcell.

## Discussion

In this study we investigated the reproduction modes of two naturally occurring supersized bacteria at the molecular level. This is the only such study besides that on the firmicute symbiont inhabiting the surgeonfish gut^13^, and the first one involving environmental sulphur-oxidizing *Gammaproteobacteria* (as shown by our 16S rRNA gene-based phylogeny). Although previous morphological studies suggested that *Eubostrichus*-associated filamentous bacteria do not divide^17^, we provided morphometric and immunocytochemical evidence that both Efs and Eds undergo binary fission. As shown by FISH with specific probes targeting their rRNA genes, only Efs and Eds are detectable on the surface of their respective hosts. Therefore, each nematode is carrying a virtually pure bacterial cell culture. This enabled us to analyze the localization pattern of the key division protein FtsZ and of the DNA in hundreds of symbiont cells. The fact that Eds appears to be the only bacterium coating its host seems to contradict a former study^20^, which suggested a high level of bacterial diversity associated with the nematode. However, all 16S rRNA gene sequences obtained in that first study originated from bacteria commonly found in marine environments; given the lack of whole nematode FISH-based evidence, these sequences likely originated from bacteria localized on nematode regions other than the cuticle (for example, the gut). Alternatively (or in addition), environmental bacteria non-stably associated with *E. dianeae* may also have been represented in the previously reported 16S rRNA gene library. Although our libraries were not extensively sequenced, they also contained non-MONTS 16S rRNA gene sequences (see Methods). Despite their heterogeneity, we could identify only a single MONTS phylotype (GenBank KF278587) and this appeared to be the only one present on the nematode cuticle by FISH. The lack of SOB sequences in the library constructed by Polz *et al*.^20^ could be due to a PCR bias, which we might have circumvented by using a shorter and more degenerate universal forward primer (616F instead of 27F). In conclusion, although we cannot exclude that bacteria other than Eds may occasionally be found on the *E. dianeae* surface, all nematode-coating bacteria visualized in our FISH analysis belong to a single MONTS phylotype. We observed the same for *E. fertilis*, suggesting high host-symbiont specificity for this newly described association as well.

The fact that there are no FtsZ rings overlapping with strong DNA signal is consistent with what is observed in *E. coli* and probably due to the phenomenon of nucleoid occlusion^32^; however, Efs and Eds DNA did not occupy the first and the last 20% of the cell length at any cell stage (Figs 4e and 5h and Supplementary Fig. 8), suggesting yet unknown mechanisms excluding DNA from the poles and preventing FtsZ polymerization in the first and the last 20% of the *Eubostrichus* ectosymbiont cells. Besides nucleoid occlusion, a second negatively acting mechanism, the Min system, dictates the selection of division sites in model bacteria^5^. In *E. coli*, the FtsZ inhibitor MinC, driven by the MinD and MinE proteins, oscillates back and forth between the two cell poles. As a consequence, MinC concentration is highest at the poles and lowest near midcell. As the cell elongates, the concentration near the cell’s centre is reduced until it becomes so low that FtsZ can polymerize and constrict the cell. Therefore, cell length is determined by the amount of MinC so that larger amounts produce longer cells^33^. The complete *min* operon is present in all the MONTS genomes sequenced so far (ref. 31 and genomic data available upon request for three additional MONTS at http://rast.nmpdr.org/rast.cgi), whereas there is no genomic evidence of another well-described FtsZ-positioning system, the DivIVA system. However, the analysis of symbiont MinC localization pattern is needed to draw conclusions about the involvement of the Min system in *Eubostrichus* ectosymbionts FtsZ-ring positioning.

Besides marine nematode *ecto*symbionts, several oversize *endo*symbionts have been described, such as legume-nodulating *Alphaproteobacteria*^34^, the gammaproteobacterium associated with *Sitophilus* weevils^35^, the bacteroidete *Sulcia* inhabiting sharpshooters^36^ and the firmicute *Epulopiscium* spp. thriving in the surgeonfish gut^37^. In the first two systems, host-secreted antimicrobial peptides (nodule-specific cysteine-rich peptides^38^ and Coleopterycin-A^39^, respectively) inhibit bacterial fission but not genome replication, which results in polyploidy. By blocking bacterial reproduction, hosts may better control the number and location of their endosymbionts^34^,^39^. The factors that trigger *Epulopiscium* sp. morphotype B extreme growth are still elusive, but polymerization of two polar FtsZ rings results in two intracellular daughter cells. These grow within the mother cell cytoplasm and are eventually released by perforating the mother cell envelope. Surprisingly, only a small portion of the mother DNA is passed on to the offspring^13^,^40^, as reviewed in ref. 41. The fragmentation of the DNA staining frequently observed in *Eubostrichus*-associated filaments suggests that they are polyploid (as is the case for all aforementioned oversize endosymbionts) and that chromosomes are segregated prior to and independently from septation. This is consistent with the hypothesis that condensation resolution should be sufficient to segregate replicated sister chromosomes before cytokinesis^42^. However, more sensitive techniques such as FISH with o*ri*C-specific probes are needed to determine the maximum genome copy numbers and the segregation dynamics in dividing Efs and Eds.

FtsZ immunostaining of *Eubostrichus*-coating bacteria revealed that, despite their large size and the likely presence of multiple genomes, they divide by symmetric transverse fission. Therefore, in contrast to all aforementioned endosymbionts that grow to extraordinary sizes because they either do not divide or they do so atypically, *Eubostrichus*-coating bacteria may become outstandingly long due to a delay in canonical binary fission. Given that Eds and Efs have not been isolated yet, we do not know whether nematode-secreted molecules mediate this delay or whether this delay is intrinsic to the bacteria. In the first case, Eds and Efs would represent the first bacteria whose growth is under host control in an *ecto*symbiosis, a form of association commonly considered less intimate than *endo*symbiosis. Besides cultivation, morphometric analysis of ectosymbionts associated with juvenile nematodes might reveal a correlation between bacterial length and host developmental stage.

It is intriguing to speculate that a similar mechanism coordinating *B. subtilis* or *E. coli* size with metabolic state (see Introduction) is at work in *Eubostrichus* symbionts. The FtsZ and DNA profiles of the crescent-shaped Efs, which are attached to its host with both poles, showed that it may divide at virtually any cell length between 3 and 45 μm. The bacterial coat is highly ordered (Fig. 1a) with the short crescent-shaped cells being proximal (that is, close to the host surface) and the long crescents being distal (Supplementary Fig. 1a,c). Their chemosynthetic metabolism relies on the diffusion of reduced sulphur compounds (for example, H_2_S) and oxygen. If we assume that the distal Efs cells have better access to them and grow faster, less nutrients will be available for the proximal ones, which will therefore grow slower. Given that Eds filaments are monopolarly attached to their host (Supplementary Fig. 1b,d), the accessibility to sulphide and oxygen does not differ among them so that they may all experience the same growth rate. The ecological advantage of the two different bacterial coat architectures remains to be determined and might be due to differences in the physiology and/or behaviour of the two *Eubostrichus* hosts.

Taken together, this first molecular study of the reproduction of two environmental *Gammaproteobacteria* showed that they may vary over 12 times in cell length, largely exceeding the size variation observed in model bacteria, and that they can divide by FtsZ-based symmetric binary fission up to a length of 120 μm. This implies that novel molecular machineries may time cell division and position the genome and division plane in *Gammaproteobacteria*, a class of several ecologically and medically important bacteria.

## Methods

### Nematode collection

Specimens of *E. fertilis* and *E. dianeae* were collected in December 2011/January 2012 and in March/April 2014 in ~1 m depth from a sand bar off Twin Cays, Belize (16° 49′ 25.74″ N, 88° 6′ 21.18″ W). The nematodes were extracted from the sand by stirring the sand in seawater and pouring the supernatant through a 63-μm-pore-size mesh sieve. The content of the net was transferred into a Petri dish and single individuals were then picked by hand using fine tweezers under a dissecting microscope. *E. dianeae* identity was assessed morphologically according to the study by Hopper *et al*.^16^ and molecularly according to the study by Kampfer *et al*.^43^ This nematode has also been referred to as *E. dianae* in the literature. To establish *E. fertilis* identity, eukaryotic 18S rRNA gene libraries were constructed using the same two gDNA extractions employed for constructing bacterial 16S rRNA gene libraries (see below). *E. fertilis* 18S rRNA gene-based phylogenetic placement is published elsewhere^15^. For gDNA extraction, Western blotting, FISH and immunostaining, nematodes were fixed in methanol and stored at −20°C for transportation and storage. For SEM, nematodes were fixed with 2.5% glutaraldehyde and stored at 4 °C.

### Scanning electron microscopy

Worms were either directly post-fixed with 1% osmium tetroxide for 2 h at room temperature, or the bacteria were first dissociated from the nematodes by mild sonication, allowed to settle on poly-L-lysine treated coverslips and then post-fixed. The samples were dehydrated in a graded ethanol series, transferred into pure acetone and critical point dried with a CPD 300 unit (Leica). After mounting, either whole worms or the bacteria-coated coverslips on stubs were gold-sputtercoated with an AGAR B7340 sputtercoater unit. Images were taken with a XL20 (Philips) using the Microscope control programme (version 7.00, FEI).

### gDNA extraction, PCR and cloning of 16S rRNA genes

For the construction of the *E. dianeae* and *E. fertilis* 16S rRNA gene libraries gDNA was extracted from two single *E. dianeae* and two single *E. fertilis* nematodes as previously described^44^. gDNA (2 μl) were used as template in each 50 μl PCR reaction. Fragments (1,499-nucleotide (nt)-long) of bacterial 16S rRNA genes were amplified by PCR with primers 616V (5′- AGAGTTTGATYMTGGCTC -3′)^45^ and 1492R (5′- GGYTACCTTGTTACGACTT -3′)^46^. Cycling conditions for the 16S rRNA gene amplification were as follows: 94 °C for 4 min followed by 35 cycles of 94 °C for 45 s, 49 °C for 30 s, 72 °C for 90 s and a final elongation of 72 °C for 10 min. We randomly picked and fully sequenced eight clones from the two *E. fertilis* 16S rRNA gene libraries. Three belonged to members of the MONTS^23^ cluster, two were from *Desulfobulbaceae*, one from *Oceanospirillales*, one from *Staphylococcus* and one from an unclassified bacterium. We randomly picked and fully sequenced 31 clones from the two *E. dianeae* 16S rRNA gene libraries. Thirteen clones were from sulphur-reducing *Deltaproteobacteria*, seven belonged to members of the MONTS cluster, three were from *Cytophaga*, five from *Rhodospirillales* and three from unclassified bacteria. Sequences were assembled with CodonCode Aligner 3.7.1 software (CodonCode Corporation, Dedham, MA, USA).

### Homology cloning of *Eubostrichus* ectosymbiont *fts*Z genes

gDNA was extracted from a single *E. fertilis* and a single *E. dianeae* as described above for the amplification of rRNA genes. For homology cloning of the Eds *fts*Z gene, a 1,106 nt-long fragment was amplified using degenerate primers ftsz1F (5′- GCVGTVATYAARGTBATCGG -3′) and ftsZ2.1R (5′- GCYGGRATRTCSAGRTAATC -3′). For homology cloning of the Efs *fts*Z gene, an additional amplification step was performed on the 1,106 nt-long ftsz1F-ftsZ2.1R fragment with the nested primer ftsZ1Fnes1 (5′- ATCAAGGTTATCGGGGT -3′). Touchdown PCR conditions for both degenerate and nested PCRs were as follows: 94 °C for 3 min, followed by 8 cycles at 94 °C for 45 s, 58–50 °C for 45 s, 72 °C for 75 s, followed by 27 cycles 94 °C for 45 s, 50 °C for 45 s, 72 °C for 75 s, and a final elongation step at 72 °C for 10 min. The Efs *fts*Z-gene fragment was directly sequenced in both directions. The Eds *fts*Z-gene fragment was cloned as described above for the rRNA genes and four clones containing the Eds *ftsZ*-gene fragment were fully sequenced in both directions. All the sequences were aligned and compared with CodonCode Aligner 3.7.1 software. We obtained a 967 nt-long sequence for Efs (GenBank accession number KF453620) and a 1.007 nt-long sequence for Eds (KF453619).

### 16S rRNA gene-based phylogenetic analysis

A bacterial 16S rRNA gene dataset was constructed by adding sequences from GenBank with >95% identity to Efs and Eds using BLASTN^47^ and selected *Chromatiaceae* as an outgroup. The sequences were aligned with MAFFT Q-INSi^48^. To reconstruct the phylogenetic relations of the symbionts we used the GTR+G+I model with maximum likelihood (RAxML^49^) and Bayesian inference (MrBayes^50^) based algorithms. To evaluate node stability we performed a rapid bootstrapping analysis (RAxML, 200 runs; ref. 51) and used posterior probabilities (MrBayes, two million generations, burnin of 25%).

### Fluorescence *in situ* hybridization (FISH)

By using the arb PROBE_DESIGN tool (the arb software package^52^; Table 2), we designed two FISH probes (Eds214 and Efs1057) specific to the SOBs identified in the *E. fertilis* and *E. dianeae* 16S rRNA gene libraries (GenBank accession numbers KF278590 and KF278587, respectively). In addition, we designed a probe (SRB64) targeting the *Deltaproteobacteria* identified in the *E. dianeae* 16S rRNA gene library (GenBank accession number KJ877189). We confirmed their specificity by comparing them with all available sequences in GenBank, the arb-silva 114 database^53^ and RDP (Ribosomal Database Project) rel.10.32 (ref. 54). The Eds214 probe has 0/3/1357 non-target hits in RDP allowing for 0/1/2 mismatches, the Efs1057 0/12/3725 and the SRB64 probe 2/493/677. All probes were fluorescently labelled on their 5′ end (Thermo Fisher Scientific, Ulm, Germany). FISH was performed on *Eubostrichus* nematodes according to the study by Manz *et al*.^55^ and as applied in the study by Bulgheresi *et al*.^25^,^26^. A detailed overview of all probes and formamide concentrations used in the different experiments is given in Table 2. To determine stringent hybridization conditions, a formamide series was conducted for all the probes (10, 15, 20, 25, 30, 35, 40 and 50%; refer to Table 2 for optimal incubation time, formamide percentage and probe concentrations). Nematodes were mounted in Vectashield (Vector Labs, Burlingame, CA, USA). When performing FISH with SRB64, bacteria were dissociated from *E. dianeae* cuticle prior to mounting. Dissociation was performed to ensure that all the bacteria attached to the cuticle would be equally prone to visual inspection, that is, to ensure that the signal emanating from putative normal-sized *Deltaproteobacteria* would not be masked by the numerically dominant, giant Eds cells. Symbiotic nematodes or dissociated symbionts were examined using a Leica TCS-SP2 confocal laser-scanning microscope combined with an inverted DM-IRE2 microscope (Leica Microsystems, Heidelberg, Germany). The EUB338 probe is from the study by Amann *et al*.^56^, Gam42a and Beta42a from the study by Manz *et al*.^55^ FISH probe names refer to their target on the *E. coli* 16S rRNA, numbering according to 57.

### Bacterial cell size and fluorescence measurements

Cells were immobilized on 1% agarose and photographed using a Leica TCS-SP2 confocal laser-scanning microscope combined with an inverted DM-IRE2 microscope (Leica Microsystems) or a Nikon Eclipse 50i microscope equipped with either a DS-Fi1 camera (Nikon) or a MFCool camera (Jenoptik). Epifluorescence images were acquired using the NIS Elements F 3.22 software (Nikon) or the ProgRes Capture Pro 2.8.8 software (Jenoptik) and processed using the public domain programme ImageJ^58^ in combination with the analysis tool Coli-Inspector^59^. Cell outlines were traced and cell length and width were measured automatically. Automatic tracing was manually double-checked and errors removed (for example, multiple cells counted as one). In the fluorescence profiles each bar represents a single bacterial cell, and the intensity of fluorescence is displayed as intensity of the respective colour (Ftsz in green and DNA in red in the overlay). The cells were sorted by increasing length from left to right. For the average fluorescence profiles, each cell was resampled to the same length and the intensities then averaged. For the fluorescence profiles, data were acquired from a single immunostaining. For the morphometric analysis and the length histograms, data from multiple experiments were pooled.

### Western Blotting, immunostaining and DNA staining

For Western blots, Efs and Eds protein extracts—obtained from dissociated ectosymbionts—were separated by reduced SDS–polyacrylamide gel electrophoresis (PAGE) on NuPAGE 4–12% Bis‐Tris pre‐cast gels (Invitrogen). They were then transferred to Hybond ECL nitrocellulose membranes (Amersham Biosciences). Membranes were blocked for 45 min in phosphate‐buffered saline (PBS) containing 5% (wt/vol) nonfat milk (PBS Milk (PBSM)) at room temperature and probed overnight at 4 °C with a commercially available rabbit polyclonal anti‐*E. coli* FtsZ antibody (Agrisera, Sweden; 1:400) in PBSM (and without, as a negative control). Unbound primary antibody was removed by three washing steps in PBSM and blots were subsequently incubated for 1 h at room temperature with a horseradish peroxidase‐conjugated anti‐rabbit secondary antibody (1:5,000; Amersham Biosciences) in PBSM. Protein–antibody complexes were visualized using ECL Plus detection reagents and films (Amersham Biosciences).

Immunostaining was performed as described^31^. Briefly, fixed nematodes were rehydrated and washed in PBS containing 0.1% Tween 20 (PBT, washing solution). Bacterial peptidoglycan was permeabilized by incubation for 20 min with 0.1% (wt/vol) lysozyme at 37 °C. Blocking was carried out for 1 h in PBT containing 2% (wt/vol) bovine serum albumin (blocking solution) at room temperature. Efs and Eds were both immunostained with a 1:200 dilution of commercially available rabbit polyclonal anti-*E. coli* FtsZ antibody (Agrisera) in blocking solution overnight under gentle agitation at 4 °C or in blocking solution alone. Unbound primary antibody was subsequently removed by three washing steps in PBT and secondary Alexa555 conjugated anti-rabbit antibody (Molecular Probes, USA) applied at a 1:500 dilution in blocking solution for 1 h at room temperature. Unbound secondary antibody was removed by three washing steps in PBT and DNA was stained with YOYO-1 Iodide (Molecular Probes) dissolved in a Tris-EDTA buffer supplemented with 50 mM potassium citrate, 0.1% Triton-X100 and 0.1 g l^−1^ of RNAseA according to the study by Marie *et al*.^60^ To dissociate Efs and Eds, worms were sonicated for 40 s in the tubes prior to mounting. Bacterial solution (1 μl) was mixed with 0.5 μl of mounting medium Vectashield (Vector Labs).

## Author contributions

The study was designed by S.B. Samples were collected by N.P, N.L., N.R.H., J.O. and S.B. DNA extraction and PCR were performed by N.P., N.L. and N.R.H. Phylogenetic analysis was performed by H.R.G.-V. Immunostaining was performed by N.P. and N.L. Image analysis was performed by N.L., T.dB. and N.P. FISH and Western blots were performed by N.P. Statistical analysis was done by N.L. Confocal microscopy was performed by S.B. The manuscript was written by S.B. and corrected by N.L, N.P, H.R.G-V., J.O. and T.dB.

## Additional information

**How to cite this article**: Pende, N. *et al*. Size-independent symmetric division in extraordinarily long cells. *Nat. Commun.* 5:4803 doi: 10.1038/ncomms5803 (2014).

## Supplementary Material

Supplementary Information Supplementary Figures 1-8 and Supplementary Table 1

## Figures and Tables

**Table 1:** Size measurements of the *Eubostrichus fertilis* and the *Eubostrichus dianeae* symbionts.

	***Eubostrichus fertilis*** **symbiont (*****n*****=2,731)**		***Eubostrichus dianeae*** **symbiont (*****n*****=2,743)**	
	**length (μm)**	**width (μm)**	**length (μm)**	**width (μm)**
Minimum	3.54	0.38	16.46	0.41
Maximum	44.55	1.21	120.22	1.40
Mean	16.23	0.60	49.70	0.75
s.d.	7.06	0.08	14.70	0.12

**Table 2:** Probes used for FISH.

**Probe**	**Specificity**	**Sequence/5′ modification**	**Target RNA**	Position*	**Formamide percentage/incubation time (h)/probe concentration (ng μl**^**−1**^)	**Reference**
EUB338	Most bacteria	5′- GCTGCCTCCCGTAGGAGT -3′Fluorescein	16S	338–355	40%/12/3.8	Amann *et al*.^56^
GAM42a	*Gammaproteobacteria*	5′- GCCTTCCCACATCGTTT -3′Cy5	23S	1,027–1,043	40%/12/2.4	Manz *et al*.^55^
BETA42a	*Betaproteobacteria*	5′- GCCTTCCCACTTCGTTT -3′Cy3	23S	1,027–1,043	40%/12/2.4	Manz *et al*.^55^
Eds214	*E. dianeae* ectosymbiont	5′- GCTCATCATCATAGCGGAA -3′Cy3	16S	214–235	40%/12/2.4	This study
Eds214mis	One mismatch to the *E. dianeae* ectosymbiont	5′- GGCTCATCATCTTAGCGGAAG -3′Cy3	16S	214–235	40%/12/2.4	This study
SRB64	Sulphate-reducing *Deltaproteobacteria* identified in the 16S rRNA gene library	5′- TGCAAGCAACCCCTTTCTCGTT -3′Fluorescein	16S	64–86	40%/12/3.8	This study
Efs1027	*E. fertilis* ectosymbiont	5′- TCACCGCGCTCCCAAGG -3′Cy3	16S	1,027–1,044	30%12/2.4	This study
Efs1027mis	One mismatch to the *E. fertilis* ectosymbiont	5′- TCACCGCGCACCCAAGG -3′Cy3	16S	1,027–1,044	30%12/2.4	This study

FISH, fluorescence *in situ* hybridization.

^*^16S rRNA position, *E. coli* numbering^57^.
