# Supplementary material for: Size-independent symmetric division in extraordinarily long cells
Source: Nat Commun. 2014 Sep 15;5:4803. doi: 10.1038/ncomms5803 (PMC4175584; doi:10.1038/ncomms5803)
Supplement: Supplementary Information — Supplementary Figures 1-8 and Supplementary Table 1 [file ncomms5803-s1.pdf]

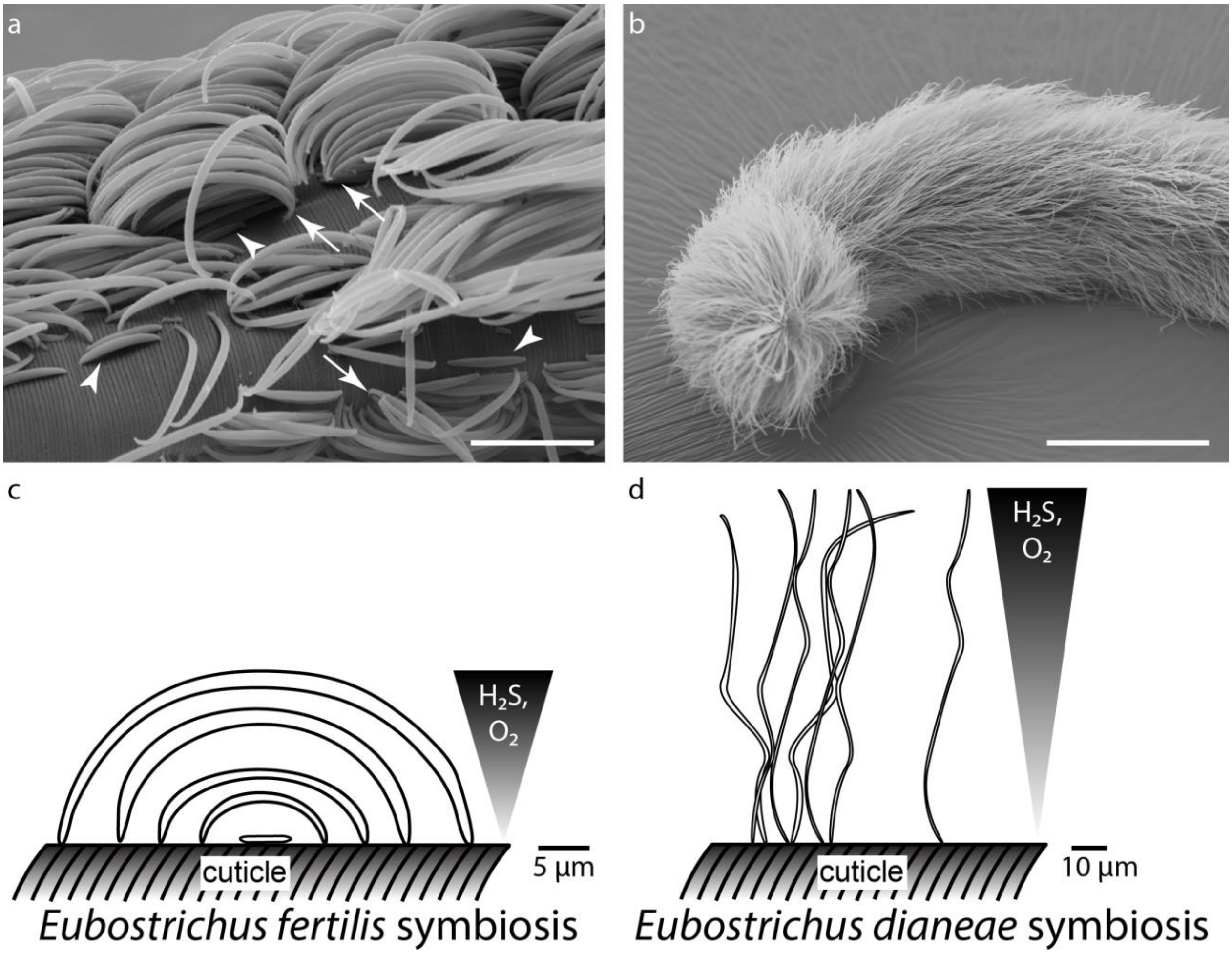

Supplementary Fig.1 The bacterial coats of *E. fertilis* and *E. dianeae*. (a) Scanning electron micrograph of the *E. fertilis* symbiosis. The bacterial coat was mechanically disturbed to show the proximal to apical arrangement of short (arrowheads) versus long crescent-shaped cells (arrows). (b) Scanning electron micrograph of the *E. dianeae* symbiosis (anterior end of the nematode). Bacterial filaments are attached with one pole to the nematode. (c-d) Sketches *of E. fertilis* and *E. dianeae* bacterial coat architectures (left and right, respectively) with proposed gradient of H_2S availability in the space between the sand and the host cuticle. Scale bar is 5 µm in (a) and 100 µm in (b).

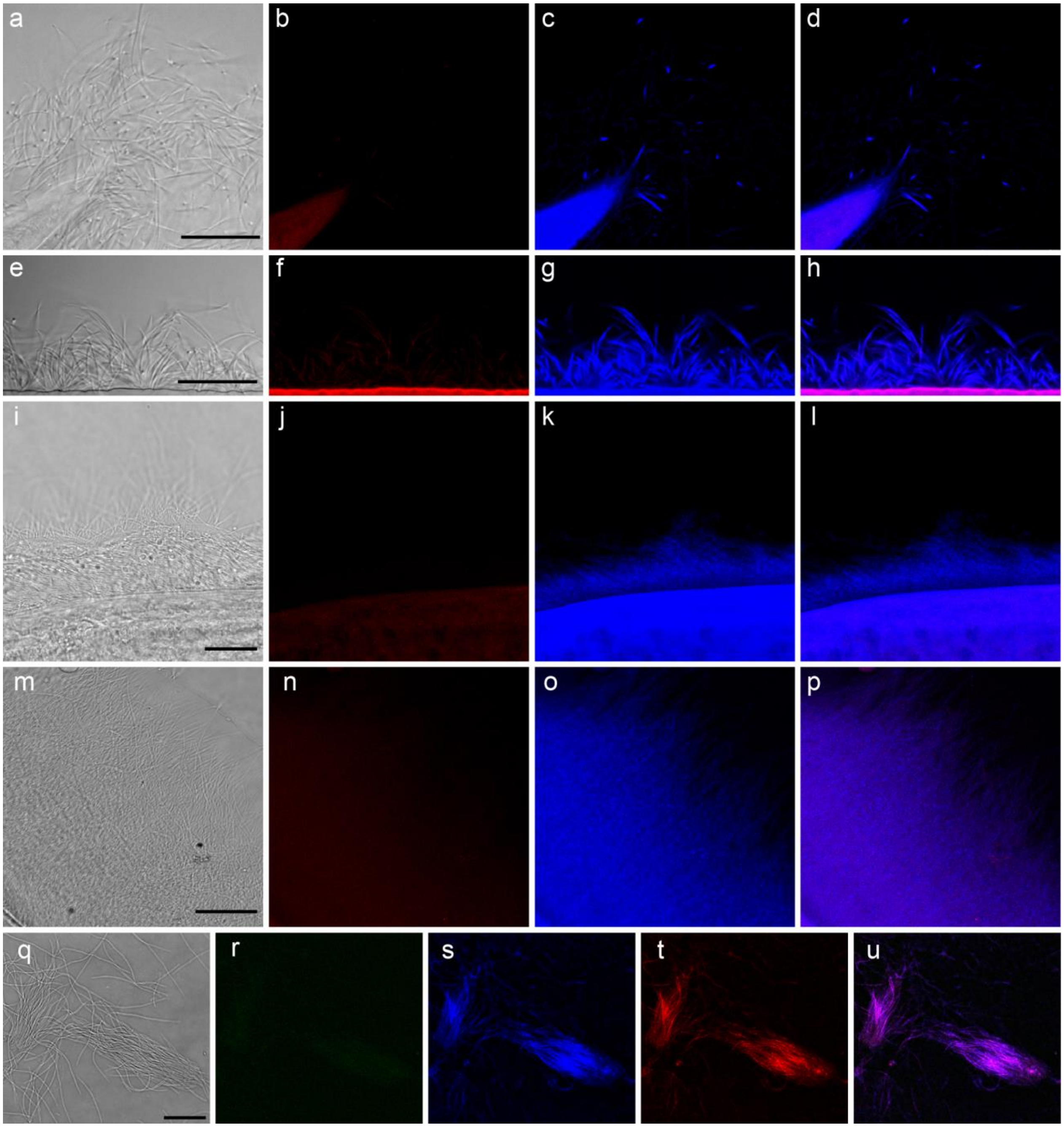

Supplementary Fig.2 FISH LSCM of *E. fertilis* (a-h) and *E. dianeae* (i-u) ectosymbionts attached to the worm surface. Bright field images (a, e, i, m) of ectosymbionts stained with a *Gammaproteobacteria*-specific probe (c, g, k, o), but neither with a *Betaproteobacteria*-specific probe (b and j) or very weakly with probes carrying a single nucleotide mismatch with the respective specific ones ((f and n), Efs1027mis and Eds214mis, respectively, see Table 1). d, h, l, and p are overlay pictures of (b-c), (f-g), (j-k) and (n-o), respectively. (q-u) shows bacteria detached from the *E. dianeae* surface that are stained with an Eds-specific probe (Eds214; (t)) and a *Gammaproteobacteria*-specific probe (s), but not with a probe targeting sulphate-reducing Deltaproteobacteria (SRB64; (r)). (q) is the corresponding bright field image and (u) the overlay of the images shown in (q-t). Scale bar is 25 μm in all images.

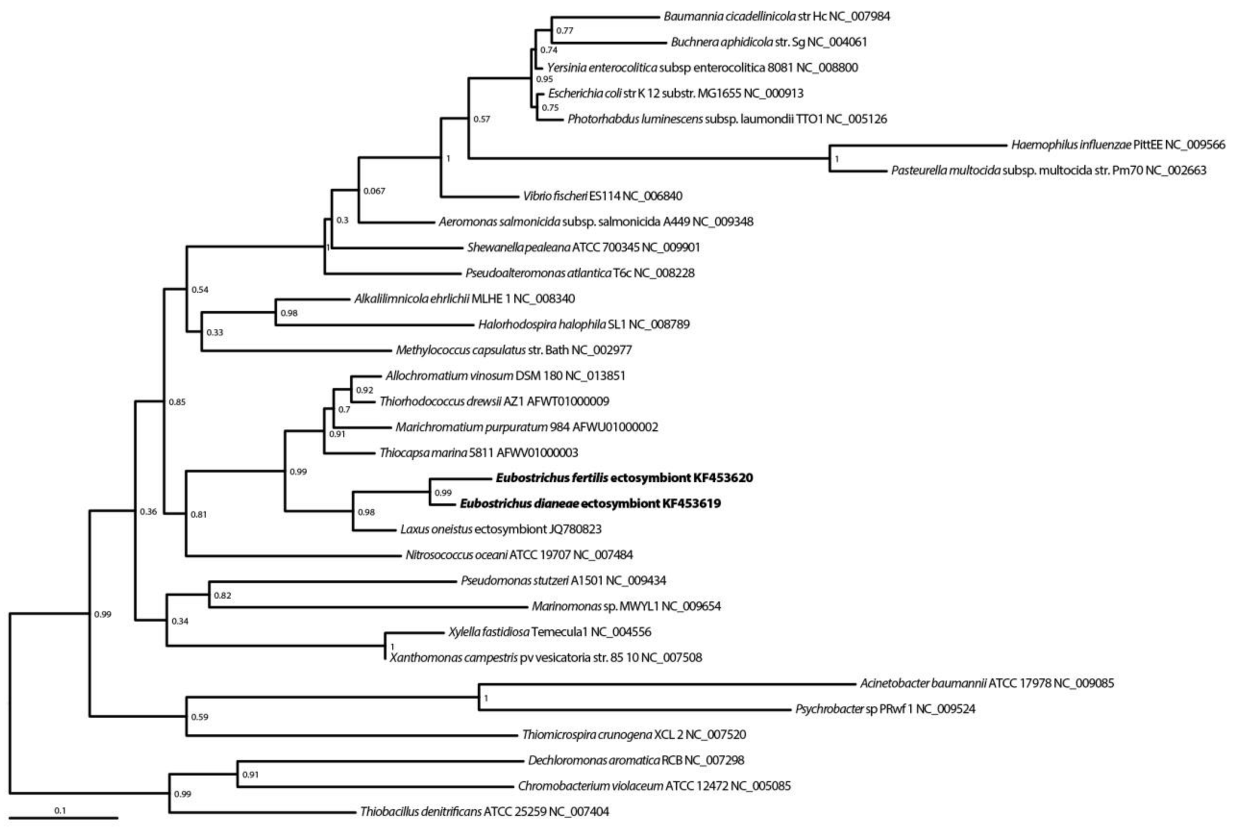

Supplementary Fig.3 Gammaproteobacterial FtsZ tree-based on the most likely PHYML tree (WAG model of substitution) using an amino acid alignment of 473 positions. aRLT node support is given for all nodes. Scale bar represents the mean number of amino acid substitutions per site. GenBank accession numbers are indicated after the names of the organisms.

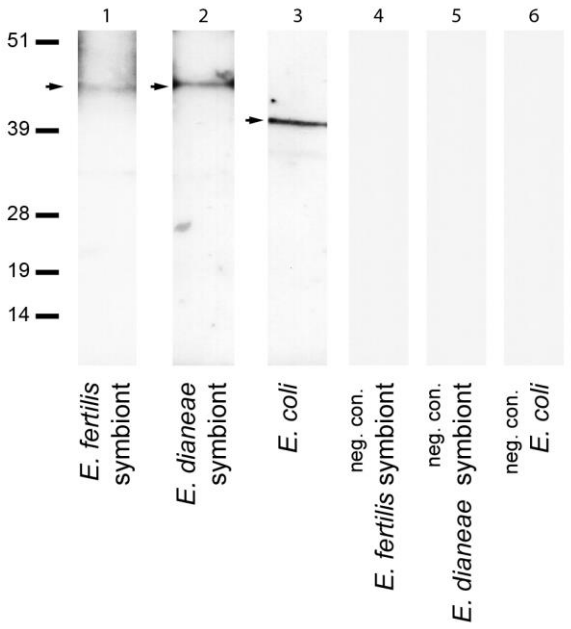

Supplementary Fig.4 Western blots of symbiont protein extracts. Efs (lanes 1 and 4), and Eds (lanes 2 and 5) and *E. coli* (lane 3 and 6) protein extracts either probed with a commercially available rabbit polyclonal anti-*E.coli* FtsZ antibody (Agrisera, Sweden; lanes 1-3) or with the secondary antibody only (lanes 4-6). Numbers indicate apparent MW expressed in kDa. Arrows indicate protein bands detected by the rabbit polyclonal anti-*E.coli* FtsZ antibody (Agrisera, Sweden).

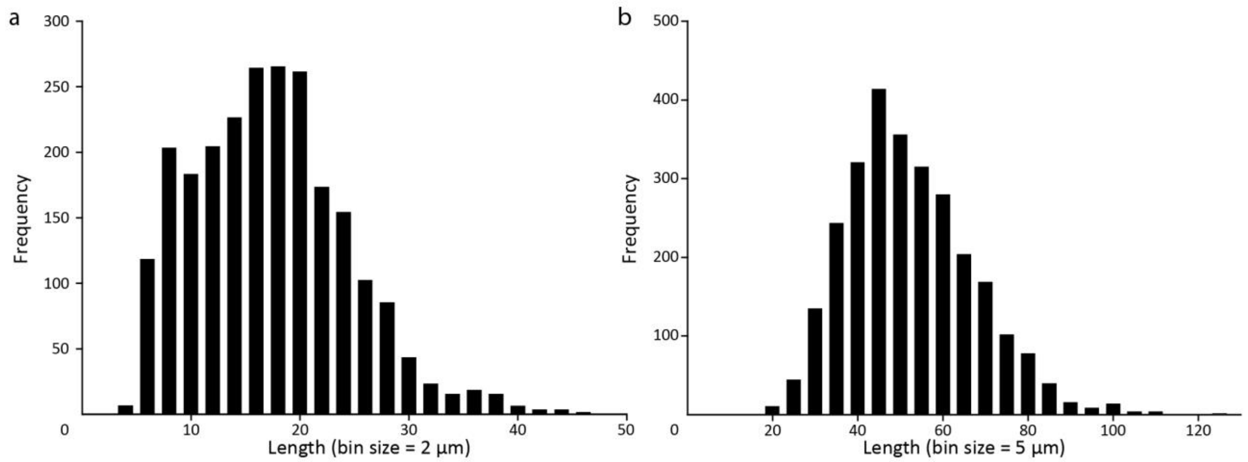

Supplementary Fig.5 Length size distributions of Efs and Eds cells. (a) Histogram showing the length size distribution of 2,371 Efs cells (bin size of 2 µm) and (b) histogram showing the length size distribution of 2,743 Eds cells (bin size of 5 µm).

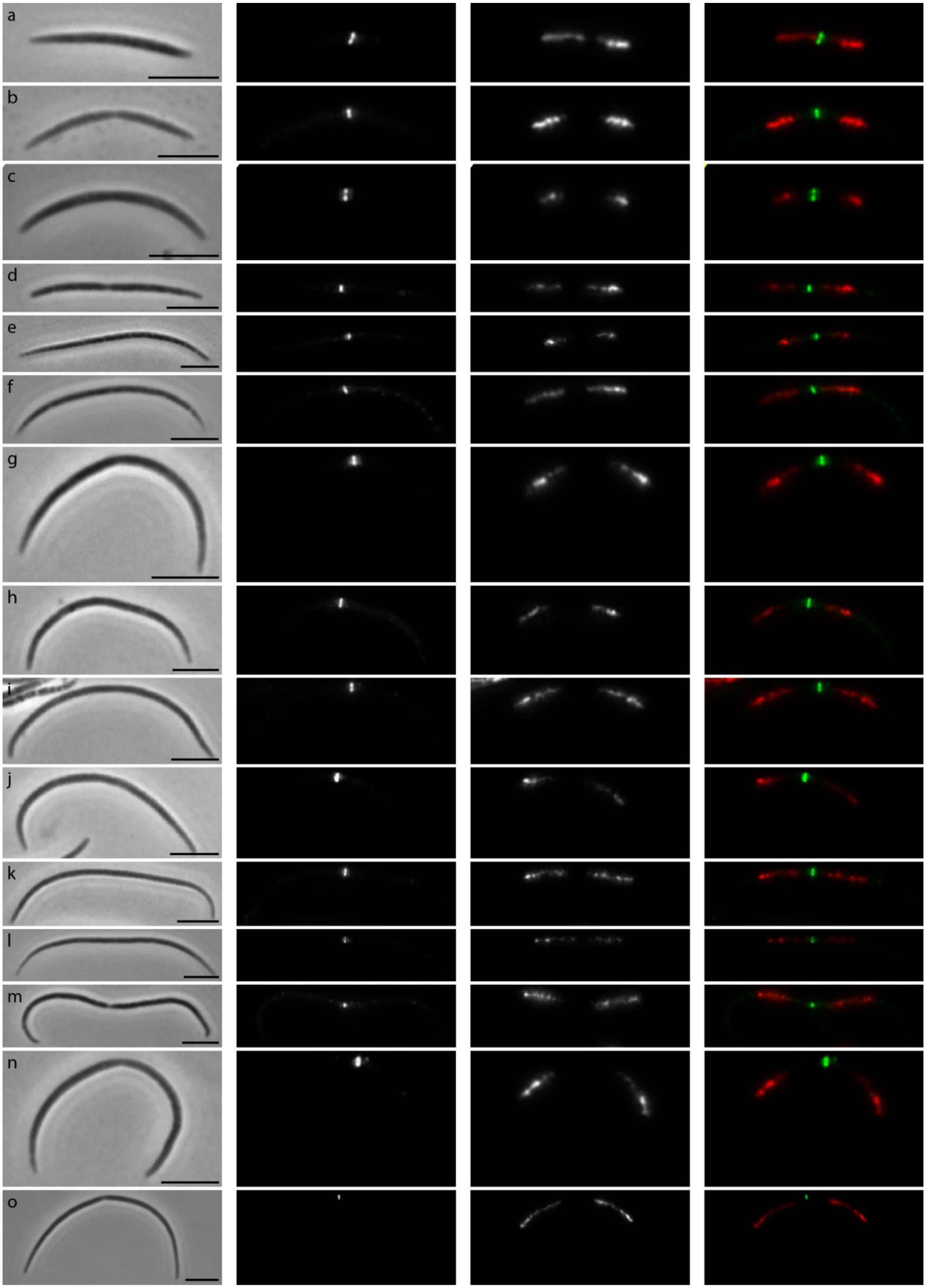

Supplementary Fig.6 FtsZ and DNA localization patterns of dividing Efs cells are shown in a-o. From left to right, phase contrast, FtsZ and DNA localization and an overlay of FtsZ (green) and DNA (red) localization. Cells are sorted by increasing length from top to bottom. Scale bar is 5 μm in each image.

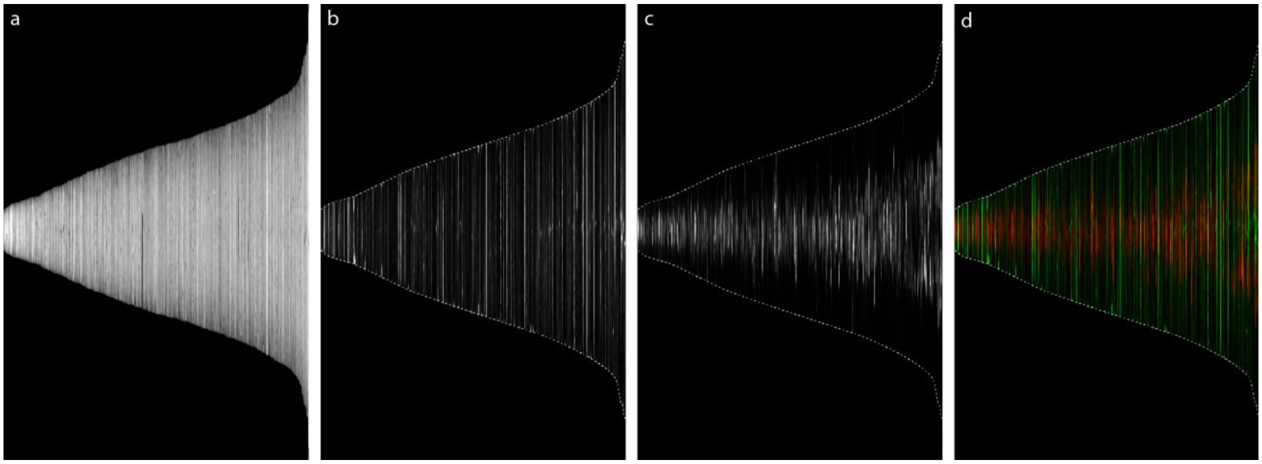

Supplementary Fig.7 FtsZ and DNA localization pattern in Efs cells. Cell length, FtsZ fluorescence, DNA fluorescence and overlay of FtsZ (green) and DNA (red) fluorescence, of 662 Efs cells are shown in (a – d) respectively. Cells are sorted by increasing cell length from left to right i.e. the leftmost bar represents the shortest and the rightmost bar the longest cell. Cell length ranges from 4.23 to 45.25 μm (mean 17.19 +/- 7.52). Dotted white line in (b-d) indicates the cell outline.

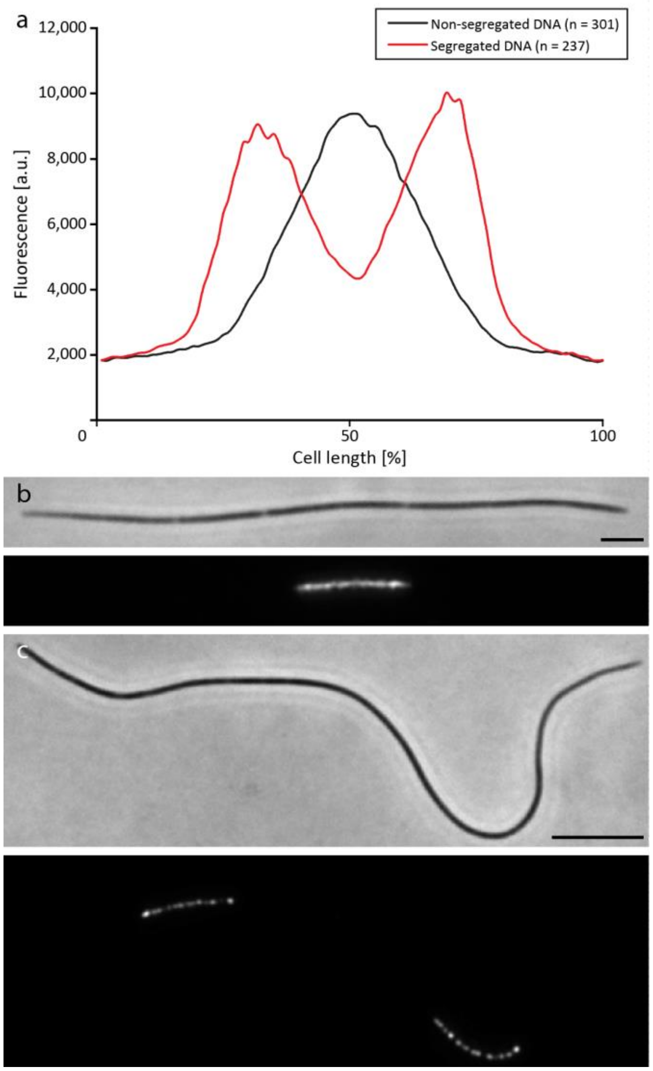

Supplementary Fig.8 DNA distribution along the Eds cell length. (a) shows an average fluorescence profile of 538 Eds cells. Black shows DNA localization in cells with non-segregated DNA (n=301) and red shows DNA localization in cells with segregated DNA (n=237) plotted along the length of the cell in percentage. (b) and (c) shows representative phase contrast and fluorescence images, from top to bottom, of cells bearing non-segregated and segregated DNA, respectively. Scale bar is 5 μm in (b) and 10 μm in (c).

Supplementary Table 1: Size distribution of cells having an FtsZ-ring.

**Length size classes of Efs cells [µm]**	**Total**	**0-15**	**15-30**	**30-45**	
# of Efs cells with a Z-ring	77	16	48	13	
% of cells with a Z-ring / size class		20.8	62.3	16.9	
**Length size classes of Eds cells [µm]**		**0-35**	**35-70**	**70-105**	**105-140**
# of Eds cells with a Z-ring	87	0	75	11	1
% of cells with a Z-ring / size class		0	86.2	12.6	1.1
